# Pharmacokinetic Studies of Triazolyl-Oxazolidinone Derivatives with Anticonvulsant Effects in Rats

**DOI:** 10.3390/pharmaceutics18101201

**Published:** 2026-09-22

**Authors:** Mohsen A. Hedaya, Mohammad G. Qaddoumi, Vidhya Thomas, Naser F. Al-Tannak, Samuel B. Kombian, Oludotun A. Phillips

**Affiliations:** 1 Department of Pharmaceutics, College of Pharmacy, Kuwait University, Safat 13060, Kuwait; vidhyaelizabeth@gmail.com; 2 Department of Pharmacology and Therapeutics, College of Pharmacy, Kuwait University, Safat 13060, Kuwait; mohammad.qaddoumi@ku.edu.kw; 3 Department of Pharmaceutical Chemistry, College of Pharmacy, Kuwait University, Safat 13060, Kuwait; dr.altannak@ku.edu.kw (N.F.A.-T.); oludotun.phillips@ku.edu.kw (O.A.P.); 4 Department of Pharmacology & Toxicology, School of Pharmacy and Pharmaceutical Sciences, University for Development Studies, Tamale NT-0272-1946, Ghana; skombian@uds.edu.gh

**Keywords:** triazolyl-oxazolidinone derivatives, anticonvulsant, pharmacokinetics, pharmacodynamics, preclinical

## Abstract

**Background/Objectives**: Five triazolyl-oxazolidinone derivatives were reported to suppress seizures in rats with different efficacies and durations of action, which may be due to different pharmacokinetic behaviors. This study sought to investigate the pharmacokinetic characteristics of the five selected triazolyl-oxazolidinone derivatives and to correlate these with their anti-seizure activity in rats. A secondary objective was to identify lead compound(s) in this small series for further modification and subsequent development into drug candidates. **Methods**: Following intraperitoneal injection, the concentrations of the five selected compounds PH066, PH139, PH162, PH166, and PH192, in plasma and brain tissues were measured using LC/MS/MS methods. The brain tissue concentrations for each derivative were subsequently correlated with its observed anti-seizure activity in male Sprague Dawley rats against electrically induced (6 Hz and MES) and chemically-induced (pentylenetetrazole) seizure models. The area under the concentration–time curves in plasma and brain tissues was determined to estimate the average brain distribution of each compound. Anti-seizure activity was quantified using a standardized behavioral scoring system based on the rat’s response to seizure-inducing stimuli, with scores assigned by two independent observers. **Results**: Plasma and brain tissue concentrations were determined over the duration of each experiment. The mean maximum plasma concentrations were 14.6, 9.20, 14.8, 18.2, and 9.00 μg/mL, while the mean maximum brain tissue concentrations were 2.68, 0.14, 1.94, 2.80, and 0.86 μg/g for PH066, PH139, PH162, PH166, and PH192, respectively. The brain to plasma distribution ratios were in the range of 0.15–0.20 for PH066, PH162, and PH166, but were much lower for PH139 and PH192. The brain tissue concentration for each compound was correlated with its anti-seizure activity. The decreasing order of anti-seizure efficacy against the 6 Hz model was PH162 > PH166 > PH066 > PH139 > PH192, which correlated well with their plasma and brain concentrations. The decreasing order of protection against the MES model was PH139 > PH192 > PH162 > PH066 > PH166, while the decreasing order of protection against the pentylenetetrazole model was PH162 ≈ PH066 > PH166 ≈ PH192 > PH139. **Conclusions**: Compounds PH066, PH162 and PH166 achieved relatively high plasma and brain tissue concentrations with good plasma to brain distribution ratios. Therefore, they have favorable pharmacokinetic characteristics when compared with compounds PH139 and PH192. Of all the compounds evaluated, PH162 had the best pharmacokinetic characteristics, with consistent moderate to high protection against all the tested seizure models and with sustained anti-seizure activity. Thus, from these studies the order of anti-seizure efficacy and duration of action is PH162 > PH139 > PH166 ≈ PH066 > PH192. Thus, PH162 emerged as the potential lead compound when both pharmacokinetic and pharmacodynamic characteristics are considered.

## 1. Introduction

The oxazolidinone scaffold is a five-membered heterocyclic ring that has been implicated as a pharmacophoric group yielding compounds with diverse pharmacological activities. Linezolid was the first member of this class of compounds approved by the USA FDA in 2000 for the treatment of resistant Gram-positive bacterial infections [1,2,3]. Since then, structurally related derivatives have been investigated for a wide variety of pharmacological activities including anti-inflammatory [4,5], antitubercular [6,7,8], antiviral [9], anticancer [10,11] and anticoagulant [12], as well as having effects in certain metabolic disorders [13,14,15] and CNS disorders such as seizures. Current epilepsy research efforts are geared towards the discovery of compounds with broad-spectrum anti-seizure activity [16,17]. Recent reviews have reported the effects of some 1,3,4-oxadiazoles [18] and 1-3-oxazolidin-2-one [19] derivatives on the central nervous system (CNS). In our laboratory we have shown that some of these triazolyl-oxazolidinone derivatives have effects on rat hippocampal cells in vitro, effects that are consistent with and suggestive of potential anti-seizure activity. We further showed that they suppressed epileptiform activity in rat hippocampal slices [20,21]. Subsequent in vivo anticonvulsant screening studies in mice, performed by the National Institute of Neurological Disorders and Stroke (NINDS) at the National Institute of Health (NIH), USA, provided functional evidence of their anticonvulsant action (Supplementary Materials). These effects in mice were generally without CNS side effects, indicating that these compounds are generally safe on brain tissue structure and function. These studies in mice were subsequently corroborated by our in vivo studies in rats, which reported the anticonvulsant effects of PH192, a triazolyl oxazolidinone (Table 1) [22]. This PH192-induced suppression of both electrically and chemically induced seizures in rats was dose- and time-dependent. However, the PH192 protection lasted for only 30 min [22].

Recently, we also investigated the anti-seizure activities of four other triazolyl-oxazolidinone derivatives, PH066, PH139, PH162, and PH166, to compare them to PH192 in terms of effects (Table 1) [23]. The selection of these analogs was based on results from the in vivo anticonvulsant screening studies in mice performed by the National Institute of Neurological Disorders and Stroke (NINDS) at the National Institute of Health (NIH), USA. The results of the screening demonstrated varied anticonvulsant activities based on the varied substituents at the piperazine N-4 position. When these compounds were administered intraperitoneally (IP) to rats at a dose of 100 mg/kg, they provided protection for the majority of rats from electrically and chemically induced seizures for at least 120 min. There were, however, differences in the efficacy and duration of anti-seizure effects among the investigated compounds [23]. We hypothesized that the possible reasons/causes of the differences in the anticonvulsant effects of these investigated compounds were differences in their pharmacokinetic characteristics. These may include their bioavailability after IP administration, the plasma–brain tissue distribution ratio that is influenced by the physicochemical properties, and the rates of elimination of the compounds, which are dependent on the efficiency of the eliminating organs such as the liver and kidneys.

The plasma and brain tissue concentrations in the samples obtained in this study were determined using recently reported, validated analytical methods [24]. Thus, the current investigation aimed to determine the pharmacokinetic characteristics of these compounds and correlate them with their anticonvulsant activity. Correlational experiments such as these are valuable in identifying the optimal structural features and favorable pharmacokinetic and pharmacodynamic characteristics of lead compounds for further development.

## 2. Materials and Methods

### 2.1. Synthesis of the Novel Triazolyl-Oxazolidinone Derivatives

The triazolyl-oxazolidinone derivatives investigated in this study are not available commercially, so they were synthetized in our laboratory according to previously reported synthetic methods [25,26,27]. The compounds were purified and their structures were fully characterized using appropriate instrumental analytical and spectral and non-spectral techniques. Dimethyl sulfoxide (DMSO), diethyl ether, pentylenetetrazol, halothane, and all the starting compounds and reagents for the synthesis of the investigated triazolyl-oxazolidinone derivatives were all obtained from Merck, Germany (formerly Sigma-Aldrich Company, Darmstadt, Germany).

### 2.2. Investigation of the Pharmacokinetics and Anticonvulsant Activities

The experiments in this study sought to evaluate the anticonvulsant activities of the selected compounds against three models of experimentally induced seizures and characterize their pharmacokinetics, including their brain concentration, in male Sprague Dawley rats. The experimental animals (325 in total) were obtained and housed at the Kuwait University HSC Animal Resource Center and were supplied with food and water ad libitum. All animal experiments were approved by the Kuwait University Health Sciences Center Animal Research Ethics committee. The experiments were performed in accordance with guidelines for the care and use of experimental animals established by the Canadian Council on Animal Care (1993 update). The procedures employed minimized animal suffering, and the minimum number of rats necessary to produce the required results was used.

#### 2.2.1. Investigation of the Pharmacokinetics and Anticonvulsant Effects Against Electrically Induced Seizures

Male Sprague Dawley rats (150–250 g; n = 225) were used in these experiments. We applied two different protocols for electrically induced seizure, and both protocols were carried out using Ugo-Basile ECT Unit 57800 (Gemonio, Italy) equipped with ear clips for rats. We have previously developed, standardized, validated, and used these models in our laboratory [22,23]. The models were the 6 Hz and the maximal electroshock (MES) models. For the 6 Hz model, seizures were induced in rats using a 40 mA current pulse, width 0.2 ms, for a total duration of 3 s, which was adapted from previously reported methods. Preliminary testing using different current strengths showed that this protocol consistently induced stage 4/5 seizures. For the MES model, seizures were induced using a 70 mA current pulse, 0.2 s duration, at 60 Hz, which was slightly modified to suit the limitations of our equipment (maximum current output 100 mA). Preliminary experiments also showed that this protocol consistently induced stage 4/5 seizures in rats. In both models, rats were considered as having seizures if they, following the electrical shock, displayed behaviors that were classified as stage 4 or 5 by two trained independent observers in accordance with classifications reported previously [28], as shown in Table 2. Additionally, based on the stage of the seizure, an anticonvulsant (protective) effect score was given to each rat, with a score of 5 given to rats showing stage 1 (no seizures) while stage 5 (full blown tonic-clonic seizures) got a protective score of 1 (Table 2).

For each of the compounds investigated in the 6 Hz model, 6 different groups of rats (n = 5 rats/group) were used. From our previous studies, we determined that the optimal dose of oxazolidinones for maximal protection was about 100 mg/kg [22]. Hence, all treated rats received 100 mg/kg of each compound under investigation dissolved in DMSO (50%) and injected IP at 15, 30, 60, 120 and 180 min prior to exposure to the 6 Hz stimulation protocol to induce seizures. In all experiments conducted in this study, control rats received an IP injection of the solvent (maximum of 1 mL of 50% DMSO). A test rat was placed in a transparent Plexiglass cage with a transparent top cover and allowed to settle in the cage prior to clamping the ears with the ear clips. The programmed stimulation protocol was then applied by a single push of a trigger button. As mentioned above, the response of each tested rat to the seizure induction was assessed independently by two trained observers. Based on the seizure stage assigned by the trained observers, each rat had an all or none response with regards to the anti-seizure activity (protected or not protected–full seizure). Immediately after the staging of seizures, the test rat was euthanized with an overdose of halothane, then blood and brain tissue samples were harvested and processed for the determination of the concentrations of the compounds. Plasma was obtained from the blood by centrifugation at 1500× *g* for 10 min. The plasma and brain tissues were weighed and kept at −80 °C until analysis.

A similar protocol was followed for studies of the MES model, except that here, 3 different groups of rats (n = 5 rats/group) for each compound were used. Rats were injected IP with 100 mg/kg of the investigated compounds dissolved in 50% DMSO and the MES protocol was applied at times of 30 and 120 min after injection, with the control rats receiving an IP injection of the solvent. The staging of seizures and harvesting of plasma and brain tissue samples followed the same methods used for the 6 Hz model.

#### 2.2.2. Pharmacokinetics and Anticonvulsant Effects Against Chemically Induced Seizures

Sprague Dawley rats (150–250 g; n = 100) were used for these experiments. Chemically induced seizures were produced by the IP injection of 75 mg/kg of pentylenetetrazol (PTZ) to rats. For each of the five compounds, four groups of rats (n = 5 rats/group) were pretreated with 100 mg/kg of the compound under investigation dissolved in 50% DMSO at 30, 60 and 120 min prior to IP injection of 75 mg/kg of PTZ. The control rats received an IP injection of the solvent (maximum of 1 mL of 50% DMSO) prior to PTZ injection. After PTZ injection, rats were placed individually in a transparent cage where they could be observed for seizure activity and staging, as described above. Cut-off time for seizure occurrence was 5 min. Immediately after staging seizures, the rats were euthanized with halothane, then blood and brain tissue were harvested for the determination of the concentrations of the compounds. Plasma was obtained by centrifugation (1500× *g* for 10 min) and tissue samples were weighed then kept at −80 °C until analysis.

### 2.3. Plasma and Brain Tissue Analysis of the Triazolyl-Oxazolidinone Derivatives

The concentrations of the investigated compounds in the plasma and brain tissue samples were determined using the LC/MS/MS methods developed and validated for each compound in our laboratories and reported previously [24]. The methods are briefly described below.

#### 2.3.1. Instrumentation

The analysis of PH066, PH139, PH162, PH166, and PH192 was performed using a Waters Acquity UPLC H-Class Xevo TQD system (Waters Corporation, Milford, MA, USA) consisting of quaternary pump, an auto-sampler, a column oven and a TQ mass spectrometer (MS/MS) equipped with an electrospray ionization probe operated in the positive ionization mode (+ESI). MassLynx software (version 3.5) was used for data processing and reporting. The chromatographic separation of analytes was carried out on an Acquity UPLC BEH C_18_ (50 mm × 2.1 mm, 1.7 um) column connected to a Van Guard Pre-column.

#### 2.3.2. Ultra-Pressure Liquid Chromatography and Mass Spectroscopy Parameters

The mobile phase consisted of filtered and degassed 0.1% formic acid in water and 0.1% formic acid in acetonitrile in various ratios—60:40, 50:50, 40:60, 50:50, and 40:60 for PH066, PH139, PH162, PH166, and PH192, respectively. The flow rate was 0.2 mL/min for all compounds, except for PH166, where the flow rate was 0.25 mL/min. The mass spectroscopy (MS) parameters were optimized for each compound to detect parent and daughter ion masses so as to enable detection and quantification.

#### 2.3.3. Sample Preparation

Samples were prepared for LC/MS/MS and UPLC according to our previously reported methods [24]. Briefly, calibration standards were prepared by spiking 200 µL blank rat plasma or brain homogenate (prepared by homogenization of brain tissues in X5 volume of phosphate buffer, pH 7.0) with 100 µL of the working solutions of compounds, producing calibration standards in the concentration range 5–30 µg/mL in plasma and 0.1–5 μg/mL in brain tissue homogenate, which is equivalent to 0.5–25 μg/g in brain tissue. Standard samples were mixed with 30 µL of 10 ng/mL methanolic solution of the IS. The mixture was vortexed for 1 min and then extracted with 600 µL of diethyl ether and centrifuged at 13,200 rpm for 10 min. The ether layer was transferred into a clean tube and evaporated to dryness under a flow of nitrogen gas. The residue was reconstituted with 300 µL of the mobile phase and filtered, and then 10 µL of the filtrate was injected onto the LC/MS/MS. When quality control samples or unknown plasma/brain were analyzed, 200 µL of sample was mixed with 100 µL of blank solvent and 30 µL of an internal standard (IS, [(*R*)-3-(3-fluoro-4-morpholinophenyl)-5-(hydroxymethyl)oxazolidin-2-one]) solution, and the resulting mixture was extracted and processed in the same way as that of the standard.

### 2.4. Pharmacokinetics and Pharmacodynamics Analyses

The concentration and effect measurements in rats that received the 6 Hz protocol were used to calculate the pharmacokinetic and pharmacodynamic parameters. The maximum plasma concentration (plasma: Cp_max_) and the maximum brain tissue concentration (brain: Cb_max_) were the highest measured concentrations in plasma and brain tissues. The area under the plasma concentration–time curve (Plasma AUC_0–180_) and the area under the brain tissue concentration–time curve (Brain tissue AUC_0–180_) during the 3 h experiment period were calculated from the measured plasma and brain tissue concentrations by the linear trapezoidal rule [29]. Furthermore, the mean anticonvulsant effect scores determined from the seizure stage, at the different time points during the 3 h experiment period, were used to calculate the area under the anticonvulsant effect score–time curve (AUE_0–180_) using the linear trapezoidal rule. The brain to plasma distribution ratio for each compound was calculated from the ratio of brain tissue AUC_0–180_ and plasma AUC_0–180_.

The brain concentration versus anticonvulsant effect (measured as the anticonvulsant activity score) relationship for each compound was evaluated in the 6 Hz protocol because of the multiple sampling times in this group of rats over a duration of 3 h. This was done by fitting the measured brain tissue concentrations and the anticonvulsant activity score that was assigned after inducing and staging seizures in individual animals to the E_max_ pharmacodynamic model using Phoenix WinNonLin v8.3, (Certara, Radnor, PA, USA) least squares regression pharmacodynamic model parameter calculation. Equation 1 describes the relationship between the compound concentration at the site of action and the compound effect in the E_max_ pharmacodynamic model.

(1) $$E=\frac{E_{max}\;C}{{EC}_{50}+C}$$

where *E* is the observed effect, *E_max_* is the maximum effect, which is the maximum anticonvulsant score in this case (equal to 5), and *EC*_50_ is the compound concentration that produces half the maximum effect, which is a measure of potency, while *C* is the compound concentration at the site of action. The assumptions of the model are that the anticonvulsant effects of these compounds are a direct effect caused by only one of the parent compounds. Also, the concentrations in the brain tissue homogenates of the compounds represent the concentrations of compounds at the sites of their anticonvulsant action. The model assumes that as the concentration of the compound increases, its effect increases until the maximum effect is achieved, at high concentrations of the compound [29].

## 3. Results

Experiments were designed such that pharmacokinetic samples were collected concurrently when performing pharmacodynamics studies. As such, several elements of the anticonvulsant activity data have been published in our earlier manuscript [23].

### 3.1. Pharmacokinetics and Anticonvulsant Effects of 6 Hz Model of Seizures

Following a 100 mg/kg IP injection of each of the triazolyl-oxazolidinone derivative or DMSO, rats were subjected to the seizure protocol at 0 (control), 15, 30, 60, 120, and 180 min. The plasma and brain tissue were harvested at these times and the concentrations of the compounds in plasma and the brain were determined (Figure 1(A1–E1); n = 5 rats per time slot). Figure 1(A2–E2) shows the anticonvulsant activities of the compounds reported as percentages of rats protected from developing seizures after the 6 Hz electrical stimulation protocol.

The plasma concentration–time profiles and the brain tissue concentration–time profiles were well characterized in this group of rats because of the multiple samples obtained over a period of 180 min after the IP administration of the compounds under investigation. The time to achieve the maximum plasma concentrations of the five compounds was within 15–30 min post-administration. The mean maximum plasma concentrations were 14.6 ± 3.58, 9.20 ± 7.1, 14.8 ± 4.1, 18.2 ± 0.78, and 9.00 ± 3.1 μg/mL (Table 3), and the mean maximum brain tissue concentrations were 2.68 ± 0.55, 0.14 ± 0.16, 1.94 ± 0.43, 2.80 ± 0.11, and 0.86± 1.20 μg/g (Table 3) for PH066, PH139, PH162, PH166, and PH192, respectively. Table 3 shows a summary of the plasma and brain tissue pharmacokinetic parameters for the five compounds.

In summary, PH162 showed excellent anticonvulsant activity in this model, demonstrated by the 100% protection of rats for up to 120 min and 80% protection of rats for up to 180 min. This was followed by PH166 and PH066, which showed very good anticonvulsant activities, producing 80% protection of rats for up to 120 min and 60% protection of rats for up to 180 min. However, PH139 had moderate anticonvulsant activities, with only about 60% protection during the entire period of the experiment, while PH192 had poor anticonvulsant activity, showing 60% protection of rats for about 30 min and 20% protection for 180 min. The three compounds, PH162, PH166, and PH066, that showed the best anticonvulsant activities had correspondingly higher plasma and brain concentrations and higher plasma to brain concentration ratios compared with PH139 and PH192, which showed moderate or poor anticonvulsant activities.

In this study, we aimed to characterize the relationship between the brain tissue concentration, which is considered the site of anticonvulsant action, and the observed anticonvulsant effect of the triazolyl-oxazolidinone derivatives under investigation in rats that received seizure-inducing stimuli. In order to determine this relationship, the E_max_ pharmacodynamics model was applied [29]. Figure 2 shows the brain concentration versus anticonvulsant activity scores of individual rats that received the 6 Hz electrical stimulation protocol after pretreatment with each of the five compounds under investigation. The pharmacodynamic model parameter estimates and the standard error of the estimates for E_max_ and EC_50_ for PH066, PH139, PH162, PH166, and PH192 are presented in Table 4. Since only one dose of 100 mg/kg IP was administered for each of the investigated compounds, the achieved brain tissue concentrations did not cover a wide range of concentrations, especially in the lower range, which is necessary for the appropriate fitting of the data to the model and the precise determination of the pharmacodynamic parameters, especially the EC_50_. Despite this limitation, it is clear from Figure 2 and the parameter estimates in Table 4 that the model used can predict, with good precision, that four of the tested compounds, PH066, PH139, PH162, and PH166, can produce anticonvulsant activity scores above 4 if the right brain concentration of the compounds is achieved. This is higher than the maximum activity score that was achieved after administration of PH192. However, the certainty of the estimates for EC_50_, which can be used as a measure of a compound’s potency, were not very good because of the limited number of low concentrations included in the fitting process.

### 3.2. Pharmacokinetics and Anticonvulsant Effects of MES Model of Seizure

Similar to the experiments above on the 6 Hz model, following the IP injections of 100 mg/kg of each of the compounds, their plasma and brain concentrations were determined at 0 (control), 30, and 120 min (Figure 3(A1–E1); n = 5 rats per time slot). At the same time, the anticonvulsant activities of the compounds were assessed and quantified as the percentage of rats protected from developing seizures after the MES electrical stimulation protocol (Figure 3(A2–E2)). From the experience gained during the 6 Hz model experiments, we decided to determine the plasma and brain concentrations and the anticonvulsant activity of the investigated compounds at early and late time slots (0, 30 and 120 min post injection) to evaluate the efficacy and duration of the anticonvulsant effect, enabling us to reduce the total number of rats used in this study (Figure 3(A1–E1,A2–E2)) without affecting the overall outcome and conclusion.

The measured plasma and brain concentrations for the five compounds at 30 min of drug administration were the highest concentrations. At 30 min after the compounds’ administration, the plasma concentrations (mean ± SE) were 9.4 ± 2.48, 1.9 ± 0.963, 23.4 ± 7.29, 18.5 ± 4.37, and 15.8 ± 1.38 μg/mL, and the mean brain tissue concentrations were 1.7 ± 0.474, 0.08 ± 0024, 2.99 ± 1.79, 1.79 ± 0.289, and 0.210 ± 0080 μg/g for PH066, PH139, PH162, PH166, and PH192, respectively. These values yielded brain to plasma concentrations ratios at 30 min of 18.0%, 4.2%, 12.8%, 9.7% and 1.3% for PH066, PH139, PH162, PH166, and PH192, respectively. These concentrations and therefore the brain to plasma ratios are similar to those reported above in the 6 Hz experiments. Again, PH066, PH162, and PH166 showed higher concentrations compared with PH139 and PH192. Interestingly, PH139, which had the lowest plasma and brain concentrations, produced 100% protection at 120 min from MES protocol-induced seizures, while PH192 produced 80% protection at 120 min. In contrast, PH066, PH162 and PH166, which achieved higher plasma and brain concentrations, produced moderate anti-seizure effects in this model, providing 60%, 60%, and 40% protection after 120 min, respectively.

### 3.3. Pharmacokinetics and Anticonvulsant Effects Against Chemically Induced Seizures

The plasma and brain tissue concentrations and the anticonvulsant activity measured as the percentage of rats protected from PTZ-induced seizure were measured at 0 (control), 30, 60 and 120 min after the IP administration of 100 mg/kg of each of the five triazolyl-oxazolidinone derivatives (Figure 4A–E). The maximum plasma and brain concentrations for the five compounds were largely achieved at 30 min after drug administration. Again, the plasma and brain tissue concentrations for the investigated compounds in the PTZ-induced seizure model were similar to the concentrations observed in the 6 Hz and MES seizure models, with higher plasma and brain tissue concentrations observed for PH066, PH162, and PH166, compared with PH139 and PH192. The maximum plasma concentrations (mean ± SE) were 11.5 ± 1.48, 8.50 ± 0.575, 13.7 ± 1.50, 19.2 ± 0.475, and 8.60 ± 1.89 μg/mL, and the maximum brain tissue concentrations were 1.7 ± 0.225, 0.1 ± 0.061, 1.5 ± 0.433, 2.1 ± 0.087, and 0.20 ± 0.132 μg/g for PH066, PH139, PH162, PH166, and PH192, respectively. These values yielded plasma to brain ratios at 30–60 min of 14.8%, 1.2%, 11.0%, 10.9% and 2.3% for PH066, PH139, PH162, PH166, and PH192, respectively. PH066 and PH162, which had moderately high plasma and brain maximum concentrations, showed an 80% protection of rats from chemically induced seizure at 120 min. While PH166, which had the highest plasma and brain maximum concentrations in this group of rats, and PH192 with a low ratio, both produced only 40% protection from the chemically induced seizures at 120 min. Finally, PH139, which had the lowest plasma and brain concentrations, produced up to 80% and 60% protection at 30 and 60 min, an effect that disappeared by 120 min. Again, this indicates that moderate brain penetrability can yield substantial protection, but pharmacokinetic processing likely makes the effect short-lived.

## 4. Discussion

This current work builds on our previously published work on PH192, and evaluations of a series of triazolyl-oxazolidinone derivatives as anticonvulsant agents [22,23]. This is part of a broader research effort to identify structural changes and physicochemical and pharmacokinetic factors that can affect the anticonvulsant activities of these compounds and identify lead compounds for further development. Our initial results for the protective effects of the studied compounds at early and late stages (120 min) show that PH066, PH139, PH162, and PH166 protected rats from electrically and chemically induced seizures for longer time periods than PH192. Because of the importance of these findings, some of the results were previously published. This includes the protective effect at 30, 60, and 120 min for all compounds from the 6 Hz protocol—Figure 3(A2–E2) and Figure 4(A2–E2). The current study continued by determining the plasma and brain concentrations of the compounds and evaluating the protective effects of the compounds from the 6 Hz protocol at five time points over a period of 180 min, and correlating the concentrations to the anticonvulsant effect. Furthermore, we determined each compound’s concentrations in plasma and brain samples obtained in the MES protocol and chemically induced seizure studies, and also correlated these with their anticonvulsant effects.

The main objective of this current work was to identify triazolyl-oxazolidinone derivatives with potential anticonvulsant effect and more favorable pharmacokinetic properties that could serve as potential lead compound(s) for further modification and/or development as anticonvulsants. Four different triazolyl-oxazolidinone derivatives were selected based on our previous in vitro and in vivo work [21,22,23], as well as the findings obtained from the U.S. NIH screening program for anticonvulsant compounds. Here, the pharmacokinetics and the in vivo anti-seizure activities of PH192 and four novel triazolyl-oxazolidinone derivatives were investigated simultaneously in rats. The correlation of the measured plasma and brain concentrations of these compounds with their anti-seizure activities could help explain the differences in the observed anti-seizure activities, as well as the durations of action among the different derivatives.

The value or importance of monitoring the compounds’ pharmacokinetic behaviors and their anticonvulsant activity simultaneously is that it allowed for the explanation of the short duration of action of PH192, which showed potential anti-seizure activity with a fairly short duration. After evaluating the plasma and brain tissue concentration–time profiles after the IP administration of PH192 obtained in the 6 Hz experiments in the current investigation, it is clear that the plasma concentrations were low, with a correspondingly very low brain tissue concentration. These low plasma and brain tissue concentrations resulted in a low brain to plasma ratio, and could explain the limited, short duration of the anticonvulsant activity of PH192. The presence of significant anti-seizure activity (60–80% protection) at such a low brain tissue concentration shortly after administration suggests that PH192 is potent and efficacious at its target(s). Similar to PH192, the other compounds investigated in the current study also produced significant anti-seizure activity at low to moderate brain concentrations. However, in contrast to PH192, these newer derivatives produced varying but longer durations of anti-seizure activities. The pharmacokinetic characteristics of these newer compounds can possibly explain the observed pharmacodynamic activities of these compounds. Therefore, studying the pharmacokinetics and pharmacodynamic effects simultaneously can be helpful in accounting for differences in observed functional activities in vivo, such as the anti-seizure activity explored here.

For both electrically and chemically induced seizures, the anti-seizure activity and plasma and brain concentrations were followed from time zero to either 120 or 180 min in groups of rats. For example, the decreasing order of anticonvulsant effect, measured as the protection imparted from 6 Hz electrically induced seizures, for the compounds under investigation is PH162 > PH166 > PH066 > PH139 > PH192. The three compounds that showed the best anti-seizure effect, PH162, PH166, and PH066, showed high plasma and brain tissue concentrations, and had the highest plasma AUC_0–180_ and brain tissue AUC_0–180_, compared with the AUCs calculated for PH192 and PH139. However, the order of the anti-seizure activities of PH162, PH166, and PH066 did not correspond to the same order for their brain tissue concentrations, as shown by the brain tissue AUC_0–180_. Thus, these three compounds may have different potencies and/or pharmacological activities due to the possible production of active metabolites, which may have been formed in vivo and contributed to the observed anticonvulsant effect. The decreasing order of the brain tissue AUC_0–180_ of the parent compounds is PH162 > PH066 > PH166 > PH192 > PH139, which is also the same order as that shown by plasma concentrations, as well as the calculated brain tissue to plasma concentration ratios (calculated from the ratio of the compound’s AUC in brain tissues to that in plasma). Furthermore, the order of Clog P for the five compounds under investigation (PH162 > PH166> PH192 > PH139 > PH066, see Table 3) was not the same as their brain to plasma concentration ratios (PH162 ~ PH066 > PH166 > PH192 > PH139, see Table 3), suggesting that the brain distribution of all the compounds may not be solely related to passive diffusion.

The brain tissue concentrations achieved were in the same range for PH066, PH162, and PH166, and were much higher than those determined for PH139 and PH192. The low brain tissue concentrations of PH192 are reflected in its relatively poor anticonvulsant effect after a longer time when compared with the other derivatives. However, compound PH139 was interesting in that, despite its very low brain tissue concentrations, it produced moderately good anticonvulsant activities for long periods. This suggests that PH139 may have high potency at its brain target(s), such that low brain concentrations produced significant protection against the tested seizure models. This resulted in an anticonvulsant activity which, although less than those shown by PH066, PH162, and PH166, was much better when compared with PH192. Although we do not yet have evidence to explain this activity at very low brain concentrations, one possibility is that PH139 may be metabolized in vivo to a metabolite that contributes to or mediates its observed anticonvulsant effect. To this end, further pharmacokinetic and pharmacodynamic investigations of PH139 are warranted to unravel the relationship of its concentration to its effects.

Looking at the structural features of the studied compounds with pronounced anti-seizure activity, we see that compounds PH139 and PH162 have a methyl group on the triazole ring. This observation suggests that methyl substitution at this position may represent a favorable structural feature within this limited series of triazolyl-oxazolidinone derivatives. The methyl group could enhance hydrophobic interactions with the molecular target and/or present appropriate physicochemical properties that could favor CNS exposure, including lipophilicity and blood–brain barrier permeability. Nevertheless, because other structural differences are present among the analogues, additional series of compounds may be required to establish the specific contributions of the triazole methyl group and other structural features around the phenyloxazolidinone scaffold to anticonvulsant activity. Also, the absence of the glycinyl space in the compound structure, such as in PH066 and PH162, seems to enhance the brain to plasma distribution ratios, which were in the range of 0.15–0.20 for PH066 and PH162. PH139 does not have the glycinyl space, but other factors such as rapid elimination may have resulted in its lower plasma and brain concentrations.

### 4.1. Comparison Across All Seizure Models

When comparing the pharmacokinetic characteristics of the various triazolyl-oxazolidinone derivatives in rats and their anti-seizure activities across the different seizure models (Figure 1, Figure 3 and Figure 4), several patterns emerge. Firstly, compounds PH066, PH162 and PH166 achieve relatively high plasma and brain tissue concentrations, and have good brain to plasma concentration ratios in the range of 0.15-2.0, which can be considered to yield more favorable pharmacokinetic characteristics compared to compounds PH139 and PH192, which have lower plasma and brain tissue concentrations. Secondly, PH162, which is one of the compounds with favorable pharmacokinetic characteristics, consistently demonstrated broad-spectrum (across all three seizure models) and sustained anti-seizure activity. It provided high protection in the 6 Hz model, robust late protection in the PTZ model, and stable efficacy in the MES model, suggesting multiple potential mechanisms of action. Thirdly, PH139 delivered rapid and potent early protection, particularly in the PTZ and MES models, but its efficacy decreased over the time tested, potentially indicating a shorter half-life and thus less favorable pharmacokinetics. Fourthly, PH166 exhibited strong early efficacy in MES and 6 Hz, but this declined substantially by 120 min in both the PTZ and MES models despite the favorable pharmacokinetic properties. Finally, compound PH066 showed modest early protection that improved at later time points in the MES test, while PH192 provided only modest, short-lived protection across all models, which can be explained by its less favorable pharmacokinetic characteristics. From the above, a suggested (decreasing) order of combined anti-seizure efficacy and duration of action emerged, as follows: PH162 > PH139 > PH166 ≈ PH066 > PH192. Thus, in the current study, PH162 emerged as the potential lead compound, providing moderate to high protection across all the models tested, with a relatively long duration of action and potentially favorable pharmacokinetics, as demonstrated by the higher plasma and brain tissue concentrations. The evidence in this study identifies PH162 as the most promising lead compound, thus making it a candidate for further structural optimization and/or development into a potential drug.

### 4.2. Limitations of Studies and Future Directions

A limitation of our experimental design is that we compared the anticonvulsant effects of the different compounds after administration of only a single dose (100 mg/kg) of each compound, rather than administering a range of doses of the different compounds. Now that we have identified a lead compound, future experiments can employ several doses. Another limitation is that in our current experiments, each animal provided one sample, as we did not perform serial time-dependent sampling because we combined the pharmacokinetic data collection with pharmacodynamics data collection, with one of the variables being seizure induction, which could not be performed repeatedly or multiple times in the same animal. It was therefore not possible to perform serial plasma sampling over time. Again, independent repeat sampling experiments need to be conducted on the lead compound to provide a full pharmacokinetic profile or full characterization of the lead compound.

Another limitation is that we only measured the concentration of the parent triazolyl-oxazolidinone compound in plasma and brain tissues. It is possible that some of these compounds may be metabolized in vivo to pharmacologically active metabolites, which could contribute to the protective effects of the compounds. Finally, this study did not perform thorough structure–activity relationship (SAR) studies of the investigated compounds. Such studies will be necessary during the optimization phase of the lead compound.

## 5. Conclusions

The present study characterized the plasma and brain pharmacokinetic profiles of five selected triazolyl-oxazolidinone derivatives, and explored the relationship between their systemic and brain exposures and antiseizure activities in rats. PH066, PH162, and PH166 achieved comparatively high plasma and brain tissue concentrations, and favorable brain-to-plasma distribution, whereas PH139 and PH192 showed substantially lower brain exposure. These pharmacokinetic differences appeared to account, at least in part, for differences in the magnitude and duration of antiseizure activities observed among the compounds, although the relationship between brain exposure and anticonvulsant effect was not consistent across all compounds and seizure models.

Among the derivatives evaluated, PH162 combined favorable pharmacokinetic characteristics with relatively consistent and sustained protection across the 6 Hz, MES, and PTZ seizure models, supporting its selection as a promising lead compound for further investigation. Future studies incorporating multiple dose levels, more comprehensive pharmacokinetic sampling, the assessment of potential active metabolites, and formal pharmacokinetic–pharmacodynamic analyses will be required to confirm the relative advantages of PH162 and guide the further optimization of this compound series.

## Figures and Tables

**Figure 1:**
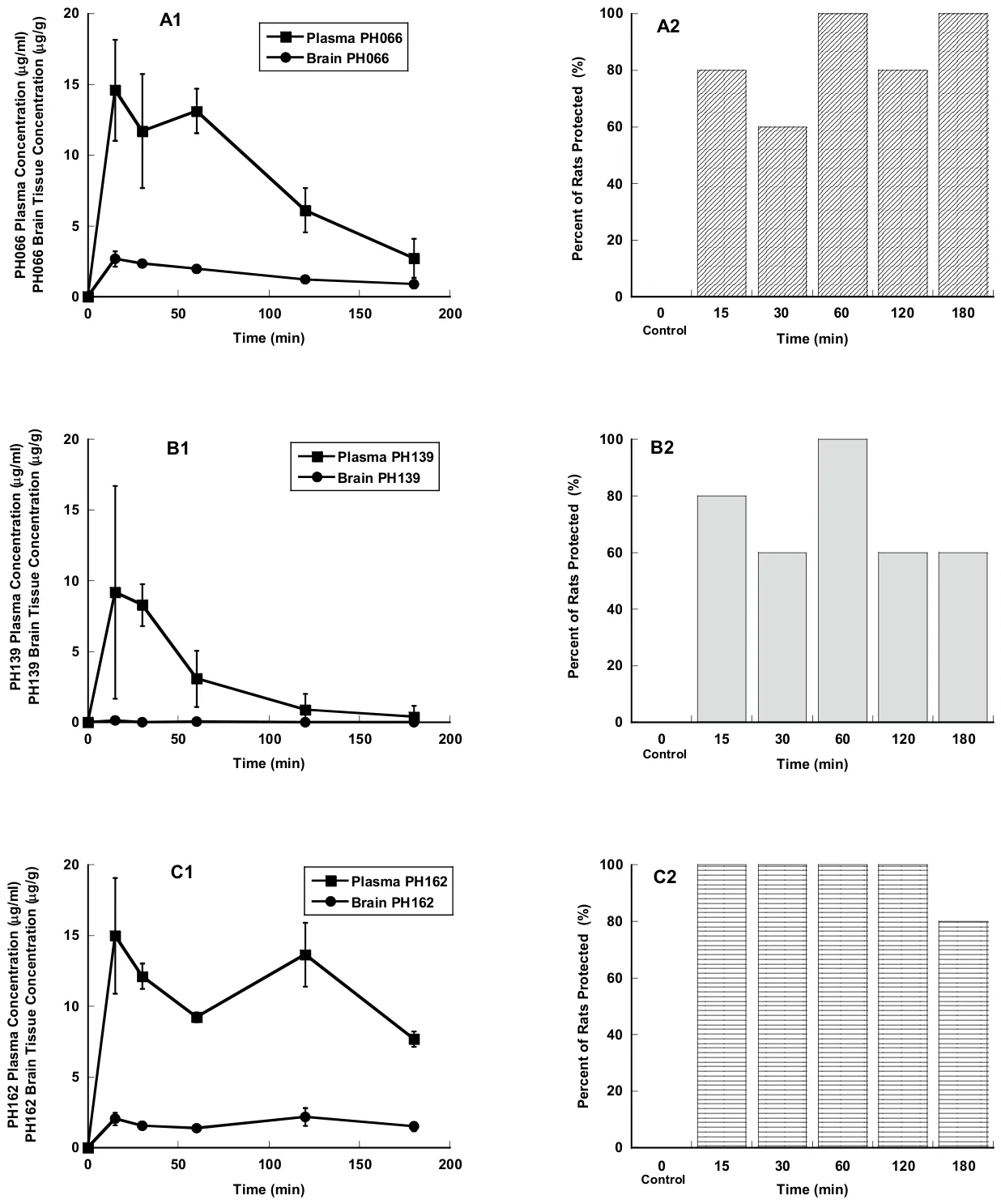

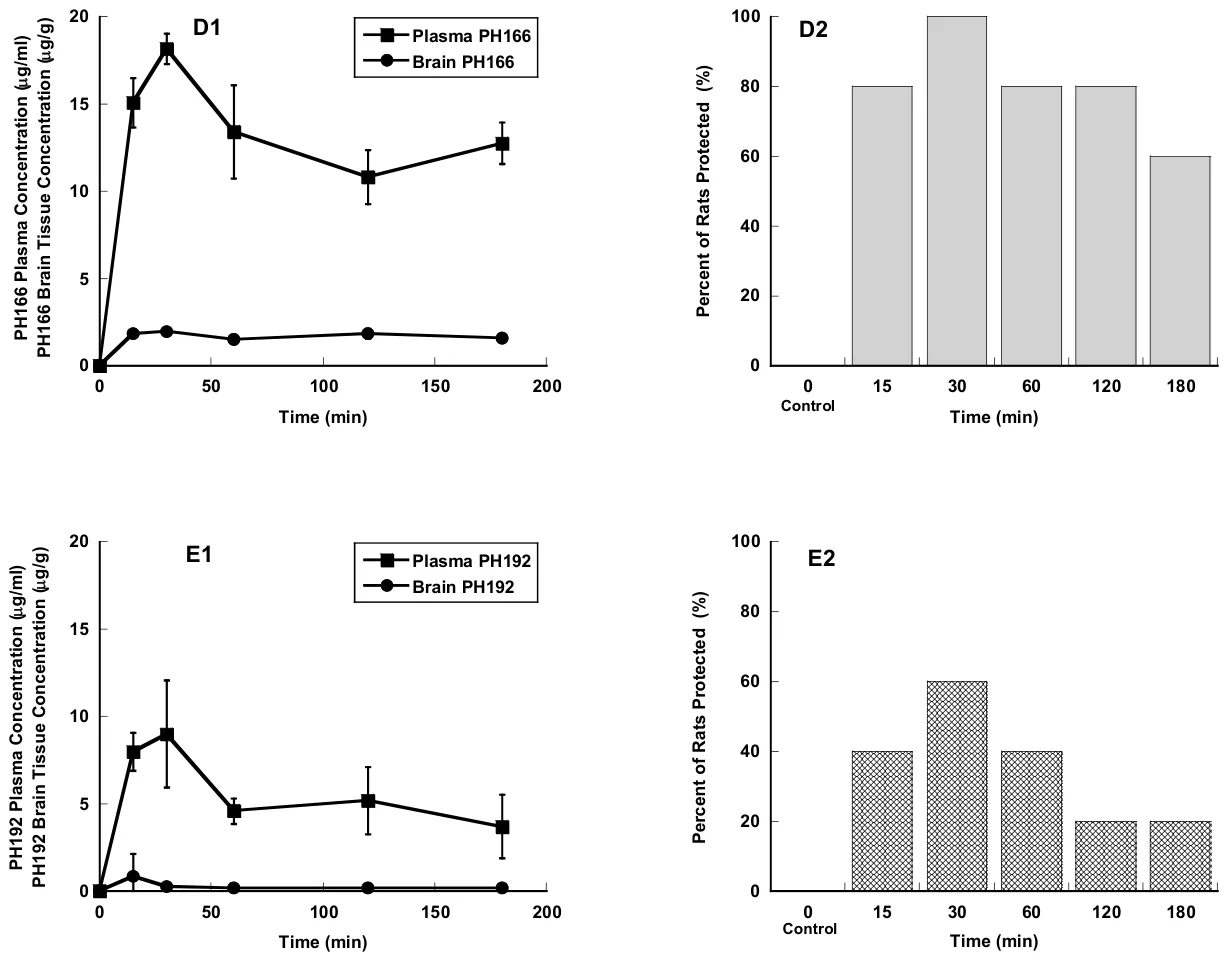
Mean ± SE (n = 5 each) of plasma and brain tissue concentration–time profiles (**A1**–**E1**), and the anticonvulsant activity measured as a percentage of rats protected from the 6 Hz electrical model of seizures (n = 5/time point) (**A2**–**E2**) for PH066, PH139, PH162, PH166, and PH192, respectively, over a period of 180 min after the IP administration of 100 mg/kg of each of the compounds. The results presented in (**A2**–**E2**) have been partially published [23].

**Figure 2:**
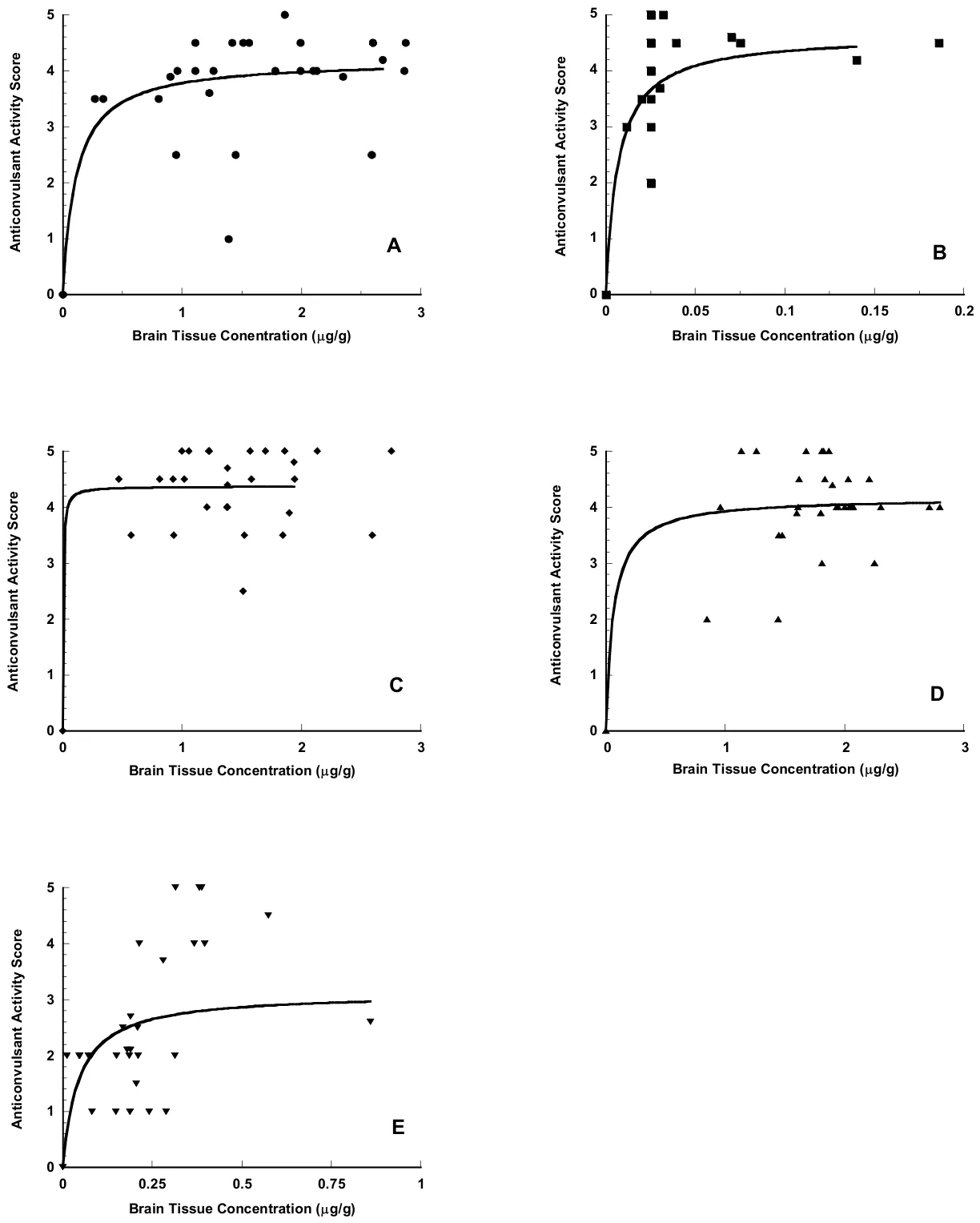
Brain tissue concentrations versus the anticonvulsant activity score in individual rats for (**A**) PH066, (**B**) PH139, (**C**) PH162, (**D**) PH166, and (**E**) 192. The symbols represent individual data points, and the solid line curves represent the E_max_ model-fitted relationship.

**Figure 3:**
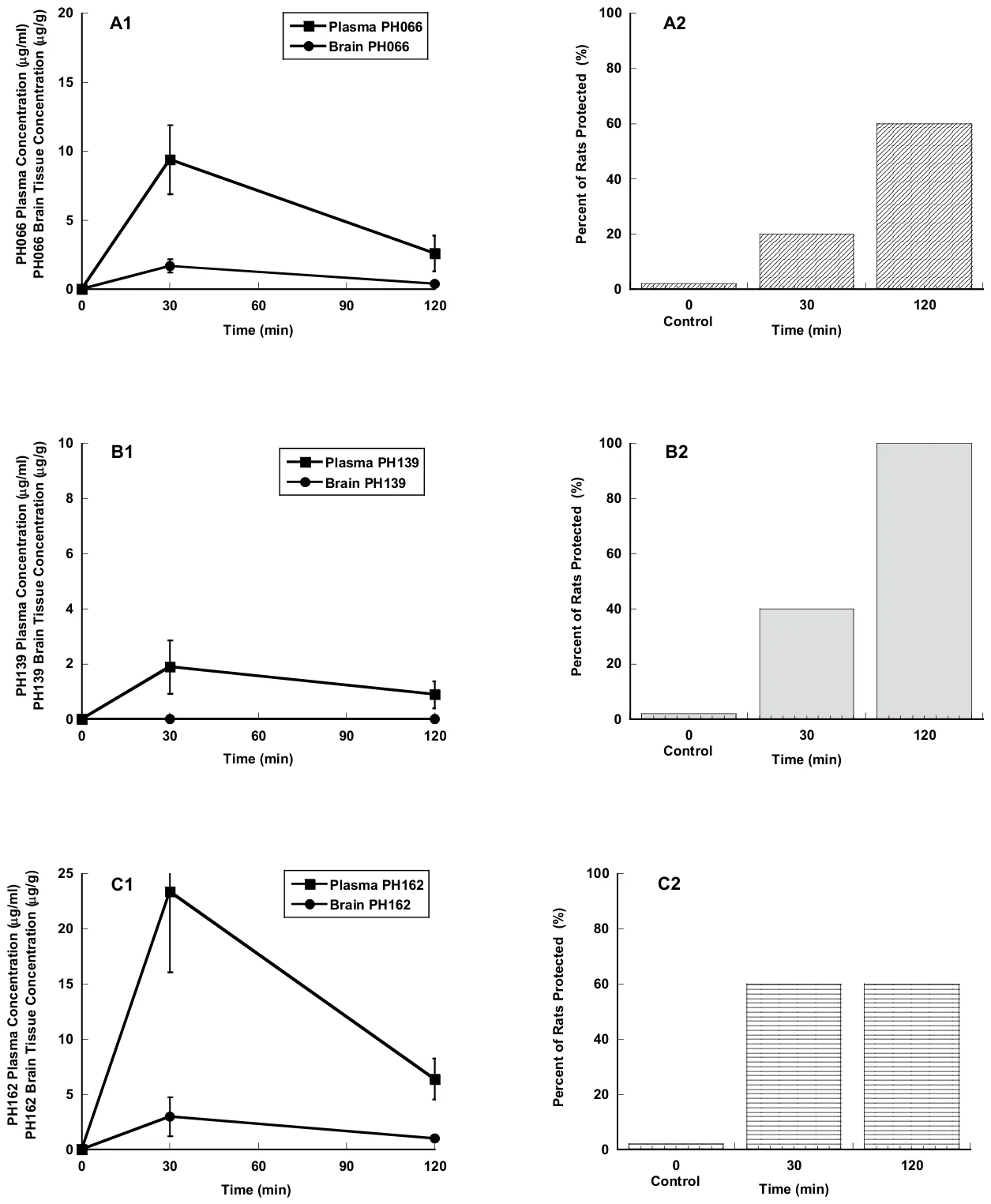

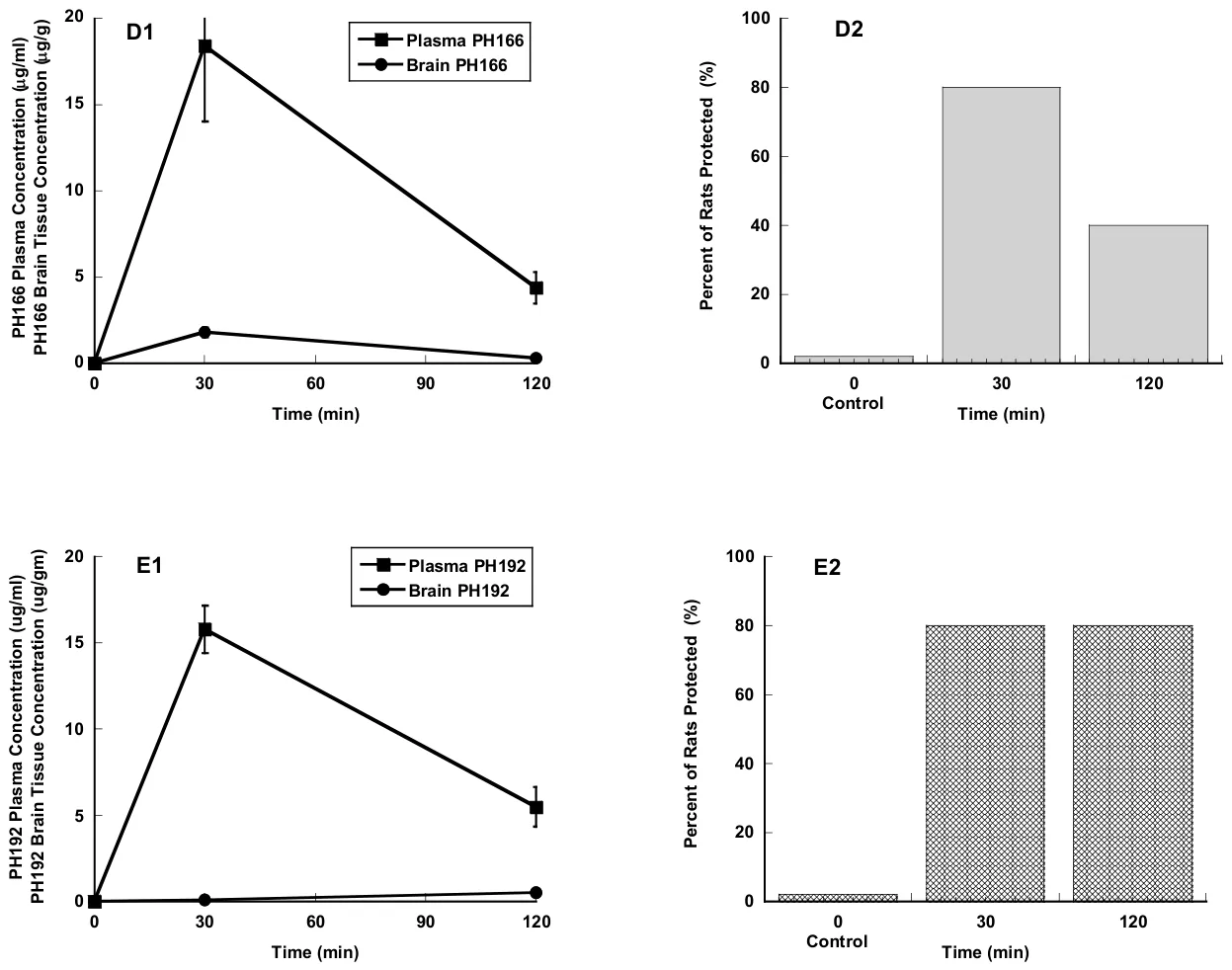
Mean ± SE (n = 5 each) plasma and brain tissue concentration–time profiles (**A1**–**E1**), and the anticonvulsant activity measured as the percent of rats protected from the MES electrical model of seizures (n = 5/time point) (**A2**–**E2**) for PH066, PH139, PH162, PH166, and PH192, respectively, over a period of 120 min after the IP administration of 100 mg/kg of each of the five triazolyl-oxazolidinone derivatives. The results presented in (**A2**–**E2**) have been published previously [23].

**Figure 4:**
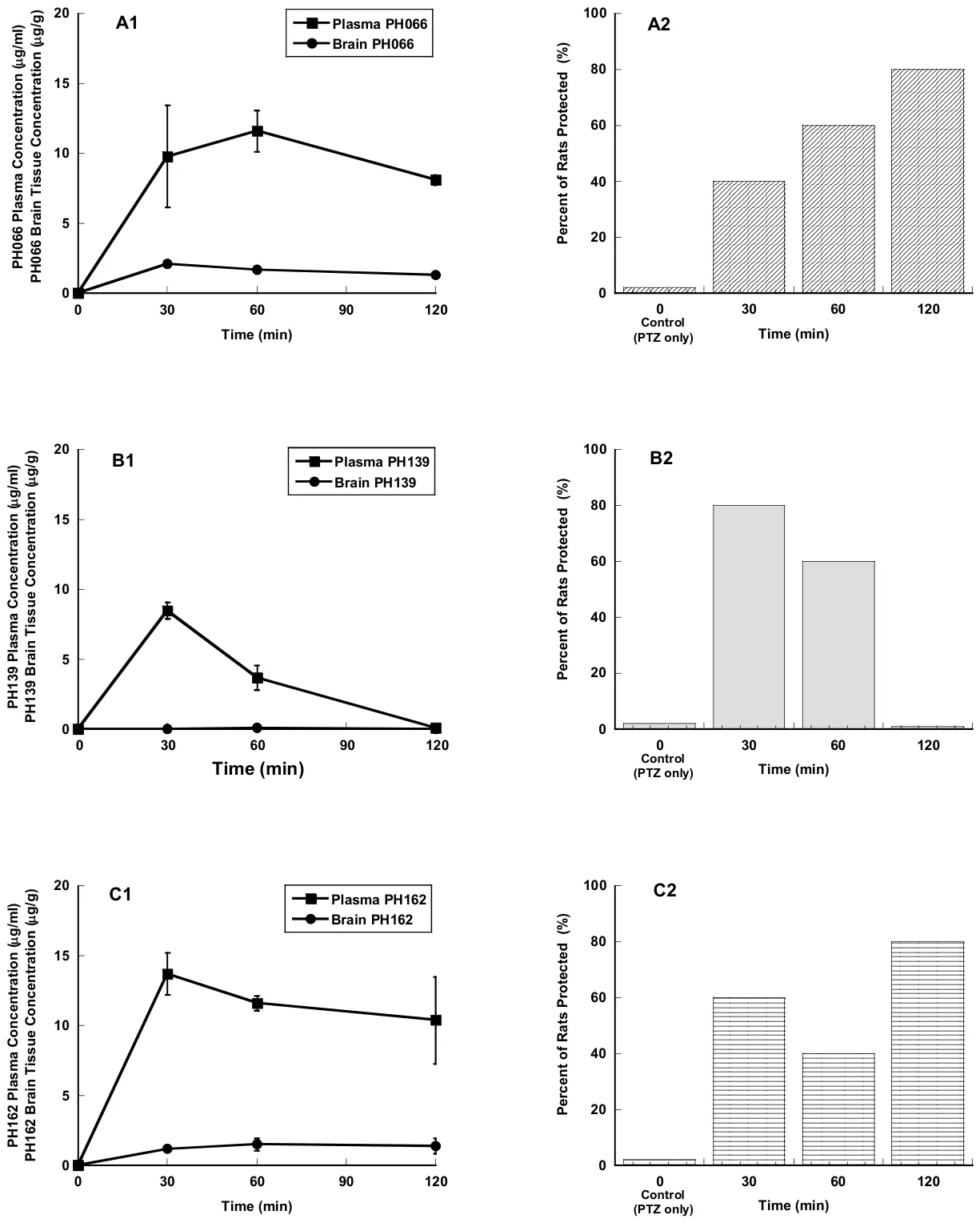

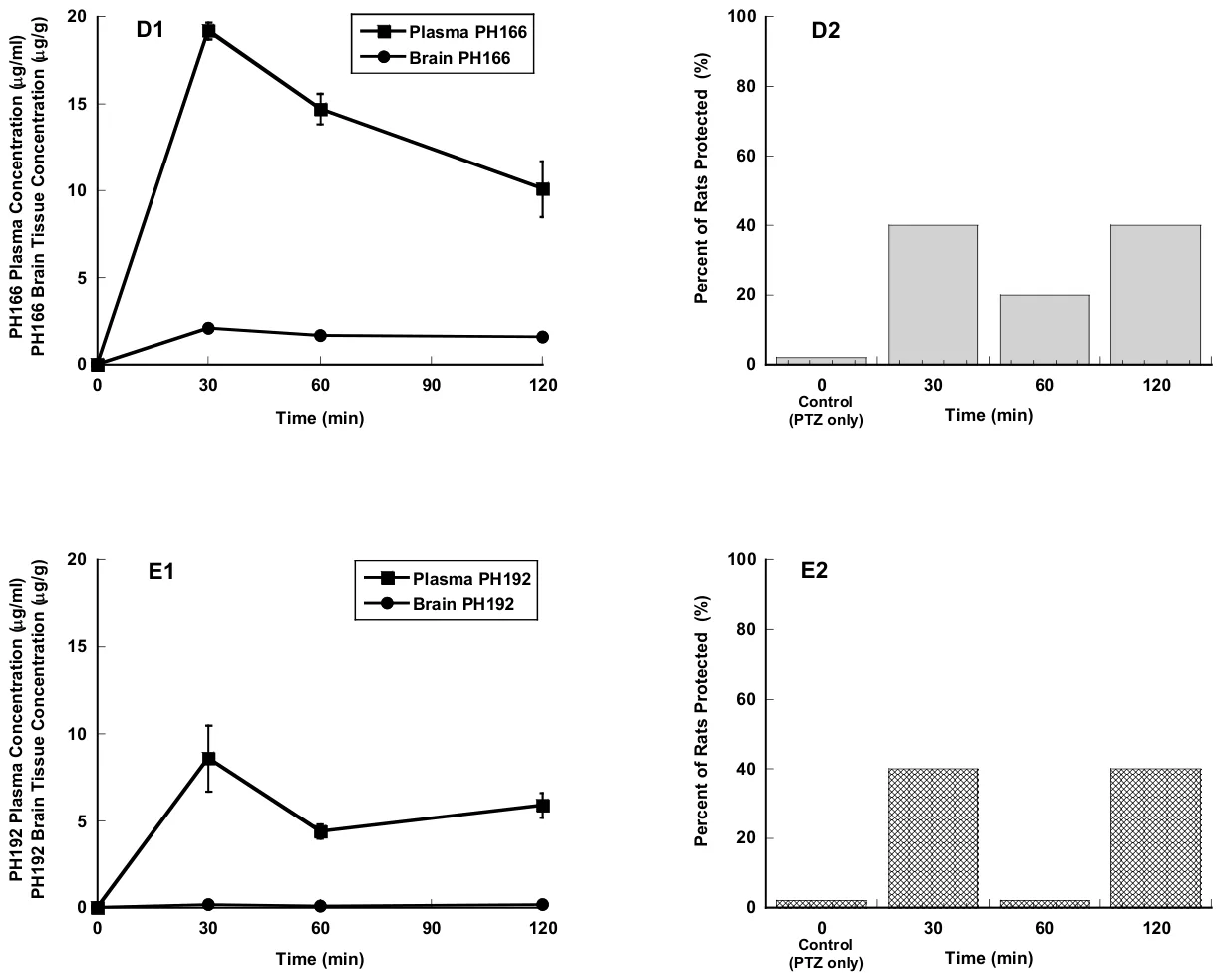
Mean ± SE (n = 5 each) plasma and brain tissue concentration–time profiles (**A1**–**E1**) and the anticonvulsant activity measured as the percent of rats protected from PTZ-induced seizures (n = 5/time point) (**A2**–**E2**) for PH066, PH139, PH162, PH166, and PH192, respectively, over a period of 120 min after IP administration of 100 mg/kg of each of the five triazolyl-oxazolidinone derivatives. The results presented in (**A2**–**E2**) have been published previously [23].

**Table 1:** Structures and molecular details of triazolyl-oxazolidinone derivatives investigated in this study.

Code #	Structures	Mol Form.	Mol wt. (Dalton)
PH066	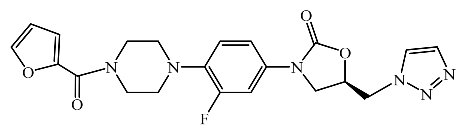	C_21_H_21_FN_6_O_4_	440.4354
PH139	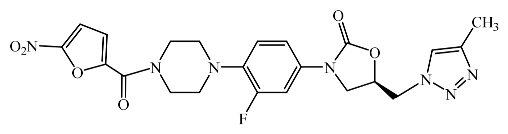	C_22_H_22_FN_7_O_6_	499.4594
PH162	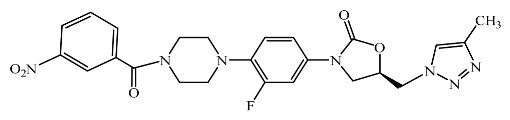	C_24_H_24_FN_7_O_5_	509.4984
PH166	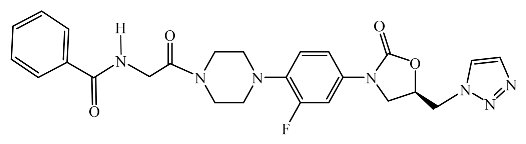	C_25_H_2_6FN_7_O_4_	507.5264
PH192	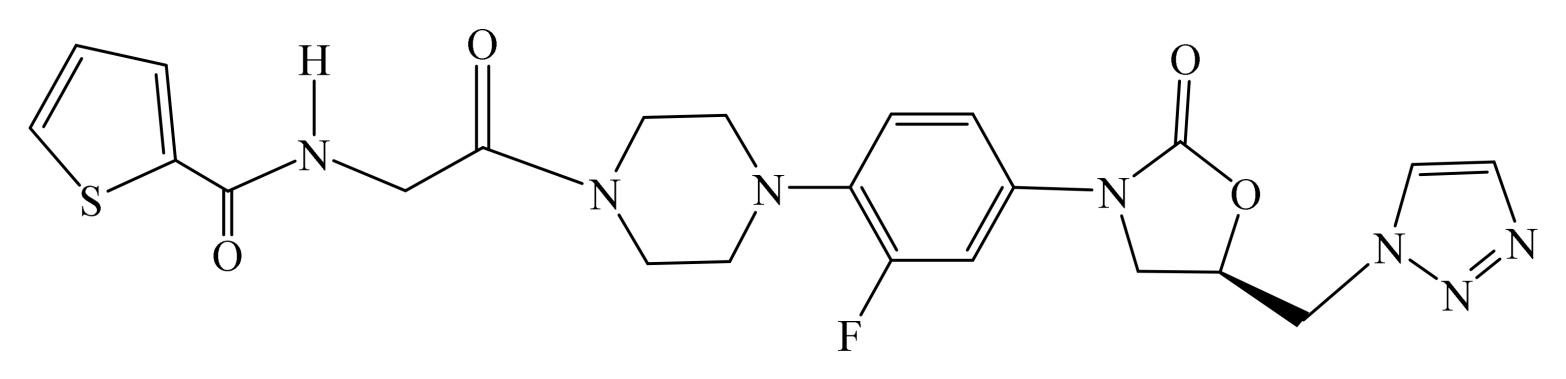	C_23_H_24_FN_7_O_4_S	513.5484

**Table 2:** Staging of in vivo seizures and the anticonvulsant effect score in rats.

Stages of Seizure	Description	Anticonvulsant Effect Score
1	Mouth and facial movement	5
2	Head nodding	4
3	Fore limb clonus	3
4	Rearing	2
5	Rearing and falling: tonic-clonic	1

Modified from Mares and Kubova [28].

**Table 3:** Summary of the plasma and brain tissue pharmacokinetic and anti-seizure parameters for the different oxazolidinone derivatives calculated from the results of the 6 Hz electrical stimulation seizure model experiments.

Compound	Plasma AUC_0–180_ μg-min/mL	Plasma Cp_max_ μg/mL Mean ± SD	Brain AUC_0–180_ μg-min/g	Brain Cb_max_ μg/g Mean ± SD	AUE_0–180_^$^ Mean Protective Response Score X min	CLog P ^#^	Brain/Plasma Distribution Ratio
PH066	1526	14.60 ± 3.58	284	2.68 ± 0.55	652	0.6088	0.186
PH139	532	9.20 ± 7.1	8.4	0.14 ± 0.16	600	0.7828	0.016
PH162	1360	14.80 ± 4.1	277	1.94 ± 0.43	833	1.6068	0.204
PH166	2272	18.20 ± 0.78	357	2.80 ± 0.11	683	1.217	0.157
PH192	951	9.00 ± 3.1	44	0.86 ± 1.20	233	1.055	0.046

^$^ Calculated by multiplying the mean protective effect score by time. ^#^ The calculated Log of partition coefficient (CLogP) values were computed using PerkinElmer ChemDraw Professional Version 19.1.1.21, PerkinElmer Informatics, Inc., Shelton, Connecticut (CT), USA 1998–2020.

**Table 4:** The estimated pharmacodynamic model parameters for the 5 different compounds under investigation.

Compound	E_max_ (SE of Estimate)	EC_50_ (SE of Estimate) (μg/g)
PH066	4.195 (0.231)	0.110 (0.088)
PH139	4.650 (0.250)	0.0068 (0.0023)
PH162	4.371 (1.180)	0.0038 (0.417)
PH166	4.170 (0.572)	0.063 (0.273)
PH192	3.120 (0.856)	0.044 (0.078)

## Data Availability

The data presented in this study are openly available from the KU Research Sector at https://www.ku.edu.kw/research (accessed on 1 August 2026).

## Supplementary Materials

The following supporting information can be downloaded at https://www.mdpi.com/article/10.3390/pharmaceutics18101201/s1, Table S1: Summary of the in vivo anticonvulsant screening studies in mice performed by the National Institute of Neurological Disorders and Stroke (NINDS) at the National Institute of Health (NIH), USA.
