# Supplementary material for: Pharmacokinetic Studies of Triazolyl-Oxazolidinone Derivatives with Anticonvulsant Effects in Rats
Source: Pharmaceutics. 2026 Sep 22;18(10):1201. doi: 10.3390/pharmaceutics18101201 (PMC13708525; doi:10.3390/pharmaceutics18101201)
Supplement: Supplementary file 1 [file pharmaceutics-18-01201-s001.zip › pharmaceutics-4508222-supplementary_ Summary of the anticonvulsant screening study.pdf]

**Table S1: Summary of the results of the *in vivo* anticonvulsant screening studies in mice performed by the National Institute of Neurological Disorders and Stroke (NINDS) at the National Institute of Health (NIH), USA.**

**Anticonvulsant Evaluation – Test 1 Results – Mice I.P. Identification, NIH**

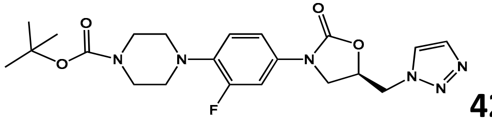


**426004 (PH036)**

| Test | Dose | Form | Dths | Time hrs. 0.25 | 0.5 | Time hrs. 1.0 | 2.0 | Time hrs. 4.0 | 6.0 | Time hrs. 8.0 | 24 |
|---|---|---|---|---|---|---|---|---|---|---|---|
| | | | | **N/F** | **N/F** | **N/F** | **N/F** | **N/F** | **N/F** | **N/F** | **N/F** |
| **MES** | 30 | SUS | | 0/1 | 0/1 | / | / | / | / | / | / |
| **MES** | 100 | SUS | | 0/3 | 0/3 | / | / | / | / | / | / |
| **MES** | 300 | SUS | | 0/1 | 0/1 | / | / | / | / | / | / |
| SC**MET** | 30 | SUS | | 0/1 | 0/1 | / | / | / | / | / | / |
| SC**MET** | 100 | SUS | | 0/1 | 0/1 | / | / | / | / | / | / |
| SC**MET** | 300 | SUS | | 0/1 | 0/1 | / | / | / | / | / | / |

| | | | | | | | | | | | |
|---|---|---|---|---|---|---|---|---|---|---|---|
| **TOX** | 30 | SUS | | 0/4 | 0/2 | / | / | / | / | / | / |
| **TOX** | 100 | SUS | | 0/8 | 0/4 | / | / | / | / | / | / |
| **TOX** | 300 | SUS | | 0/4 | 0/2 | / | / | / | / | / | / |

# Anticonvulsant Evaluation – Test 1 Results – Mice I.P. Identification, NIH

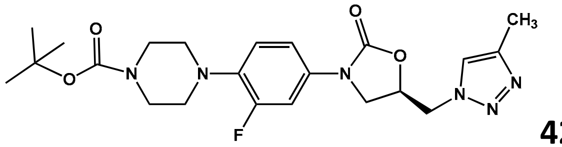


**426005 (PH108)**

| **Test** | **Dose** | **Form** | **Dths** | **Time hrs. 0.25** | **0.5** | **Time hrs. 1.0** | **2.0** | **Time hrs. 4.0** | **6.0** | **Time hrs. 8.0** | **24** |
|---|---|---|---|---|---|---|---|---|---|---|---|
| | | | | **N/F** | **N/F** | **N/F** | **N/F** | **N/F** | **N/F** | **N/F** | **N/F** |
| **MES** | 30 | SUS | | 0/1 | 0/1 | / | / | / | / | / | / |
| **MES** | 100 | SUS | | 0/3 | 0/3 | / | / | / | / | / | / |
| **MES** | 300 | SUS | | 0/1 | 0/1 | / | / | / | / | / | / |

| | | | | | | | | | | | |
|---|---|---|---|---|---|---|---|---|---|---|---|
| SC**MET** | 30 | SUS | | 0/1 | 0/1 | / | / | / | / | / | / |
| SC**MET** | 100 | SUS | | 0/1 | 0/1 | / | / | / | / | / | / |
| SC**MET** | 300 | SUS | | 0/1 | 0/1 | / | / | / | / | / | / |
| **TOX** | 30 | SUS | | 0/4 | 0/2 | / | / | / | / | / | / |
| **TOX** | 100 | SUS | | 0/8 | 0/4 | / | / | / | / | / | / |
| **TOX** | 300 | SUS | | 0/4 | 0/2 | / | / | / | / | / | / |

# Anticonvulsant Evaluation – Test 1 Results – Mice I.P. Identification, NIH

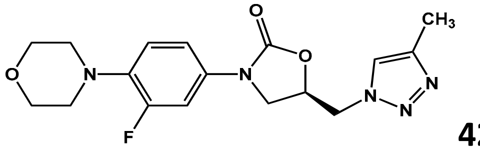


**426006 (PH084)**

| **Test** | **Dose** | **Form** | **Dths** | **Time hrs.** | | **Time hrs.** | | **Time hrs.** | | **Time hrs.** | |
|---|---|---|---|---|---|---|---|---|---|---|---|
| | | | | **0.25** | **0.5** | **1.0** | **2.0** | **4.0** | **6.0** | **8.0** | **24** |
| | | | | **N/F** | **N/F** | **N/F** | **N/F** | **N/F** | **N/F** | **N/F** | **N/F** |
| **MES** | 30 | SUS | | 0/1 | 0/1 | / | / | / | / | / | / |
| **MES** | 100 | SUS | | 0/3 | 0/3 | / | / | / | / | / | / |
| **MES** | 300 | SUS | | 0/1 | 0/1 | / | / | / | / | / | / |

| | | | | | | | | | | | |
|---|---|---|---|---|---|---|---|---|---|---|---|
| SC**MET** | 30 | SUS | | 0/1 | 0/1 | / | / | / | / | / | / |
| SC**MET** | 100 | SUS | | 0/1 | 0/1 | / | / | / | / | / | / |
| SC**MET** | 300 | SUS | | 0/1 | 0/1 | / | / | / | / | / | / |
| **TOX** | 30 | SUS | | 0/4 | 0/2 | / | / | / | / | / | / |
| **TOX** | 100 | SUS | | 0/8 | 0/4 | / | / | / | / | / | / |
| **TOX** | 300 | SUS | | 0/4 | 0/2 | / | / | / | / | / | / |

## Anticonvulsant Evaluation – Test 1 Results – Mice I.P. Identification, NIH

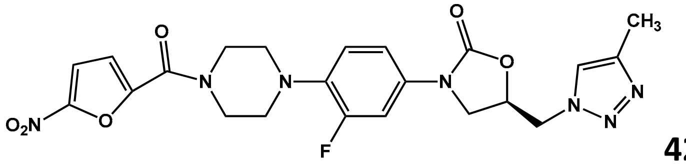


**426007 (PH139)**

| **Test** | **Dose** | **Form** | **Dths** | **Time hrs.** 0.25 | 0.5 | **Time hrs.** 1.0 | 2.0 | **Time hrs.** 4.0 | 6.0 | **Time hrs.** 8.0 | 24 |
|---|---|---|---|---|---|---|---|---|---|---|---|
| | | | | **N/F** | **N/F** | **N/F** | **N/F** | **N/F** | **N/F** | **N/F** | **N/F** |
| **MES** | 30 | SUS | | 0/1 | 0/1 | / | / | / | / | / | / |
| **MES** | 100 | SUS | | 0/3 | 0/3 | / | / | / | / | / | / |
| **MES** | 300 | SUS | | 0/1 | 0/1 | / | / | / | / | / | / |

| | | | | | | | | | | |
|---|---|---|---|---|---|---|---|---|---|---|
| SC**MET** | 30 | SUS | 0/1 | 0/1 | / | / | / | / | / | / |
| SC**MET** | 100 | SUS | 0/1 | 0/1 | / | / | / | / | / | / |
| SC**MET** | 300 | SUS | 0/1 | 0/1 | / | / | / | / | / | / |
| **TOX** | 30 | SUS | 0/4 | 0/2 | / | / | / | / | / | / |
| **TOX** | 100 | SUS | 0/8 | 0/4 | / | / | / | / | / | / |
| **TOX** | 300 | SUS | 0/4 | 0/2 | / | / | / | / | / | / |

# Test 7 - Results - Anticonvulsant Evaluation (6Hz, Mice), of Oxazolidinones - NIH

| **ADD number** | **Compd ID** | **Structure** | **Test** | **Dose** | **Time hrs. 0.25** | **0.5** | **Time hrs. 1.0** | **2.0** | **Time hrs. 4.0** | **6.0** | **Time hrs. 8.0** | **24** |
|---|---|---|---|---|---|---|---|---|---|---|---|---|
| | | | | | **N/F** | **N/F** | **N/F** | **N/F** | **N/F** | **N/F** | **N/F** | **N/F** |
| **426004** | **PH036** | 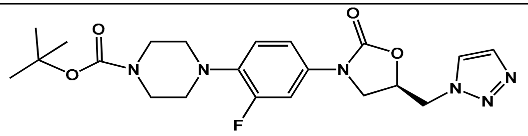 | 6Hz | 100 | 0/4 | 0/4 | 0/4 | 0/4 | 0/4 | / | / | / |
| | | | Tox | 100 | 0/4 | 0/4 | 0/4 | 0/4 | 0/4 | / | / | / |
| **426005** | **PH108** | 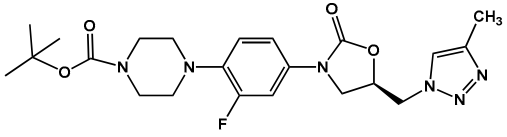 | 6Hz | 100 | 0/4 | 0/4 | 0/4 | 0/4 | 0/4 | / | / | / |
| | | | Tox | 100 | 0/4 | 0/4 | 0/4 | 0/4 | 0/4 | / | / | / |
| **426006** | **PH084** | 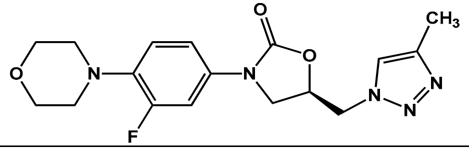 | 6Hz | 100 | 0/4 | 0/4 | 0/4 | 0/4 | 0/4 | / | / | / |
| | | | Tox | 100 | 0/4 | 0/4 | 0/4 | 0/4 | 0/4 | / | / | / |

| | | | | | | | | | | | | |
|---|---|---|---|---|---|---|---|---|---|---|---|---|
| **426007** | **PH139** | 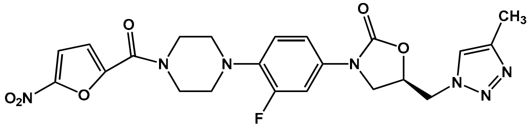 | 6Hz | 100 | **1/4** | **2/4** | **2/4** | **2/4** | **1/4** | **/** | / | / |
| | | | Tox | 100 | **0/4** | **/4** | **0/4** | **0/4** | **0/4** | **/** | / | / |
| **429019** | **PH065** | 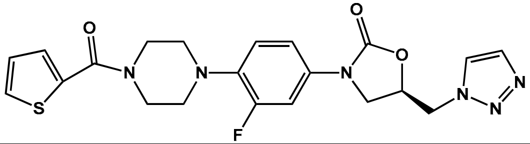 | 6Hz | 100 | **0/4** | **1/4** | 0/4 | 0/4 | 0/4 | / | / | / |
| | | | Tox | 100 | **0/4** | **0/4** | 0/4 | 0/4 | 0/4 | / | / | / |
| **429020** | **H066** | 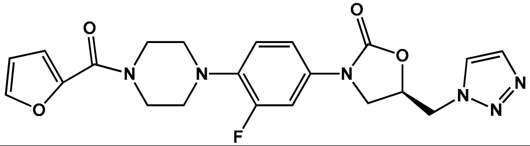 | 6Hz | 100 | 0/4 | 0/4 | **3/4** | **0/4** | 0/4 | / | / | / |
| | | | Tox | 100 | 0/4 | 0/4 | **0/4** | **0/4** | 0/4 | / | / | / |
| **429021** | **PH050** | 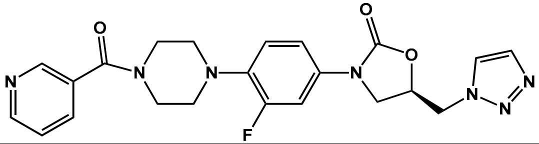 | 6Hz | 100 | 0/4 | 0/4 | 0/4 | 0/4 | 0/4 | / | / | / |
| | | | Tox | 100 | 0/4 | 0/4 | 0/4 | 0/4 | 0/4 | / | / | / |

# Test 7 - Results - Anticonvulsant Evaluation (6Hz, Mice), of Oxazolidinones - NIH

| **ADD number** | **Compd ID** | **Structure** | **Test** | **Dose** | **Time hrs. 0.25** | **0.5** | **Time hrs. 1.0** | **2.0** | **Time hrs. 4.0** | **6.0** | **Time hrs. 8.0** | **24** |
|---|---|---|---|---|---|---|---|---|---|---|---|---|
| | | | | | **N/F** | **N/F** | **N/F** | **N/F** | **N/F** | **N/F** | **N/F** | **N/F** |
| **429022** | **PH144** | 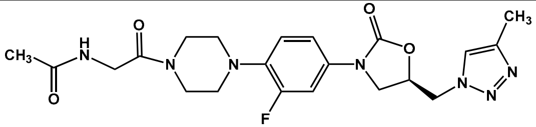 | 6Hz | 100 | 0/4 | 0/4 | 0/4 | 0/4 | 0/4 | / | / | / |
| | | | Tox | 100 | 0/4 | 0/4 | 0/4 | 0/4 | 0/4 | / | / | / |
| **429023** | **PH146** | 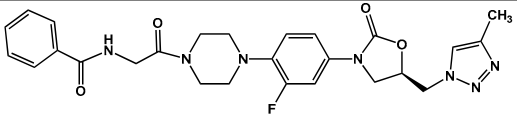 | 6Hz | 100 | **2/4** | 0/4 | 0/4 | 0/4 | 0/4 | / | / | / |
| | | | Tox | 100 | **0/4** | **0/4** | 0/4 | 0/4 | 0/4 | / | / | / |
| **429024** | **PH148** | 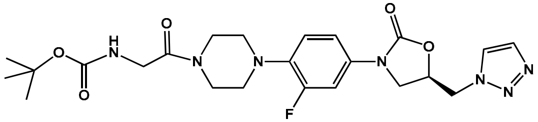 | 6Hz | 100 | 0/4 | 0/4 | 0/4 | 0/4 | 0/4 | / | / | / |
| | | | Tox | 100 | 0/4 | 0/4 | 0/4 | 0/4 | 0/4 | / | / | / |

| | | | | | | | | | | | | |
|---|---|---|---|---|---|---|---|---|---|---|---|---|
| **429025** | **PH153** | 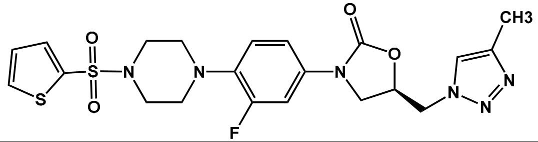 | 6Hz | 100 | **1/4** | **3/4** | **1/3** | **0/4** | 0/4 | / | / | / |
| | | | Tox | 100 | **0/4** | **0/4** | **0/4** | **0/4** | 0/4 | / | / | / |
| **429026** | **PH154** | 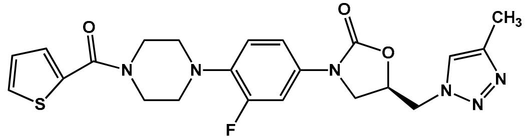 | 6Hz | 100 | 0/4 | 0/4 | 0/4 | 0/4 | 0/4 | / | / | / |
| | | | Tox | 100 | 0/4 | 0/4 | 0/4 | 0/4 | 0/4 | / | / | / |
| **29027** | **PH157** | 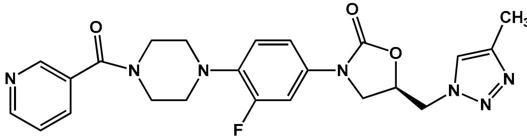 | 6Hz | 100 | 0/4 | 0/4 | **1/4** | 0/4 | 0/4 | / | / | / |
| | | | Tox | 100 | 0/4 | 0/4 | **0/4** | **0/4** | 0/4 | / | / | / |
| **429028** | **PH162** | 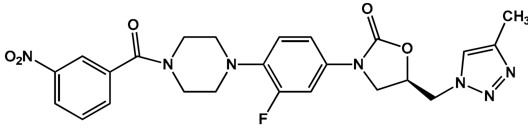 | 6Hz | 100 | **2/4** | **2/4** | **2/4** | **1/4** | 0/4 | / | / | / |
| | | | Tox | 100 | **0/4** | **0/4** | **0/4** | **0/4** | 0/4 | / | / | / |

# Test 7 - Results - Anticonvulsant Evaluation (6Hz, Mice), of Oxazolidinones - NIH

| ADD number | Compd ID | Structure | Test | Dose | Time hrs. 0.25 | 0.5 | Time hrs. 1.0 | 2.0 | Time hrs. 4.0 | 6.0 | Time hrs. 8.0 | 24 |
|---|---|---|---|---|---|---|---|---|---|---|---|---|
| | | | | | **N/F** | **N/F** | **N/F** | **N/F** | **N/F** | **N/F** | **N/F** | **N/F** |
| **429029** | **PH166** | 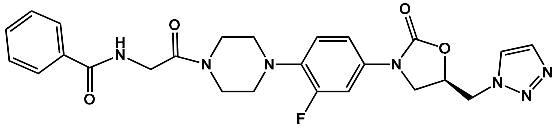 | 6Hz | 100 | **1/4** | **1/4** | **2/4** | 0/4 | 0/4 | / | / | / |
| | | | Tox | 100 | **0/4** | **0/4** | **0/4** | **0/4** | 0/4 | / | / | / |
| **429030** | **PH175** | 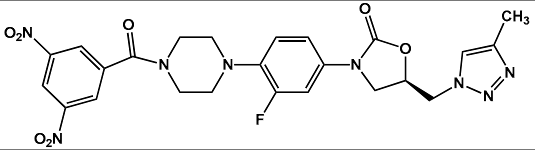 | 6Hz | 100 | **1/4** | **0**/4 | 0/4 | 0/4 | 1/4 | / | / | / |
| | | | Tox | 100 | **0/4** | **0/4** | **0/4** | **0/4** | **0/4** | **/** | / | / |

| | | | | | | | |
|---|---|---|---|---|---|---|---|
| **429031** | **PH192** | 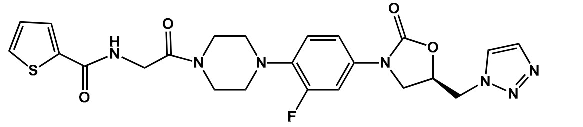 | 6Hz 100 | **2/4** **3/4** | **1/4** 0/4 | 0/4 / | / / |
| | | | Tox 100 | **0/4** **0/4** | **0/4** **0/4** | 0/4 / | / / |

N = responded (protected) F = total number of animals used
